# Metabolites in Early‐Mid Pregnancy Mediate the Association Between Prepregnancy Body Mass Index and Risk of Gestational Diabetes Mellitus

**DOI:** 10.1155/jdr/6303241

**Published:** 2026-02-04

**Authors:** Suna Wang, Yanwei Zheng, Mingjuan Luo, Wei Chen, Jingyi Guo, Rongzhen Jiang, Xiangtian Yu

**Affiliations:** ^1^ Clinical Research Center, Shanghai Sixth People’s Hospital Affiliated to Shanghai Jiao Tong University School of Medicine, Shanghai, China, sjtu.edu.cn; ^2^ Department of Obstetric and Gynaecology, Shanghai Sixth People’s Hospital Affiliated to Shanghai Jiao Tong University School of Medicine, Shanghai, China, sjtu.edu.cn; ^3^ Department of Endocrinology and Metabolism, The University of Hong Kong-Shenzhen Hospital, Shenzhen, China, hku-szh.org

**Keywords:** gestational diabetes mellitus, mediation analysis, metabolomics, prepregnancy body mass index

## Abstract

**Aims:**

The study is aimed at identifying the shared metabolites in early‐mid pregnancy associated with prepregnancy body mass index (pBMI) and subsequent gestational diabetes mellitus (GDM) risk and at exploring the mediating role of metabolites.

**Methods:**

One hundred pregnant women with GDM and 100 matched controls were enrolled in the study. Serum samples were collected in 10–20 weeks’ gestation and used for targeted metabolomic assay measurement. The associations among pBMI, metabolites, and GDM were investigated using linear regression and logistic regression models. Mediation analysis was conducted to evaluate the mediating effect of individual metabolite and clustered latent variable (LV) on the association of pBMI with GDM.

**Results:**

We identified eight metabolites significantly associated with both pBMI and GDM, which contained three organic acids, three acylcarnitines, and two fatty acids. Mediation analysis found five individual metabolites and two clustered LVs exhibited significant mediation effects in the association between pBMI and GDM risk. LV1 showed mediated proportions of 24.0%, which represented as organic acids and enriched in branched‐chain amino acid biosynthesis. LV2 showed mediated proportions of 19.1%, which represented as acylcarnitines and enriched in linoleic acid metabolism. Furthermore, we validated the mediating role of branched‐chain amino acids during the OGTT period.

**Conclusion:**

The association between pBMI and GDM risk was attributed to serum metabolites in early‐mid pregnancy, especially metabolites related to branched‐chain amino acid biosynthesis.

## 1. Introduction

Gestational diabetes mellitus (GDM), defined as hyperglycemia first detected in pregnancy, is a common pregnancy complication [1]. The occurrence of GDM is associated with a higher risk of adverse pregnancy outcomes and future Type 2 diabetes and cardiovascular disease in both mother and offspring [2, 3]. Several risk factors have been proven to increase GDM, among which prepregnancy body mass index (pBMI) showed the strongest contribution [4]. Although existing evidence suggests excess insulin resistance and insufficient insulin secretion may be the key pathophysiology linking pBMI and GDM, the underlying mechanism was complex and remained unclear [5, 6]. This knowledge gap limits targeted preventions and interventions, as pBMI alone fails to account for metabolic heterogeneity among pregnant women.

Metabolomics was an effective methodology systematically depicting metabolite profile, which enables a comprehensive understanding of metabolic changes in disease progression, especially for metabolic diseases. A wide range of previous studies have revealed significant metabolite change during pregnancy for either pBMI or GDM, indicating the potential of metabolomic profiling to elucidate biochemical pathways linking maternal adiposity to glucose dysregulation [7–9]. For example, one metabolomic research has found higher pBMI altering maternal early‐pregnancy amino acids, nonesterified fatty acids, phospholipids, and carnitines, and longitudinal metabolomics has reported that amino acid and its derivatives were significantly associated with GDM across three trimesters [9, 10]. However, the findings of differential metabolites have been inconsistent, and few studies have systematically explored their metabolic interrelationships between pBMI and GDM. Furthermore, pBMI has an impact on metabolic processes during the early stages of pregnancy, which leads to potential metabolic disturbances prior to the diagnosis of GDM, ultimately facilitating the subsequent onset [11, 12]. There is a lack of evidence regarding metabolite profiles in early pregnancy that explain the development of GDM induced by pBMI [7, 11, 13].

In this study, we conducted a case–control study to investigate the shared serum metabolic profile for both pBMI and GDM in pregnancy with serum samples collected in 10–20 weeks’ gestation. We then performed mediation analyses to explore whether the shared metabolites or latent variables (LVs) derived from these metabolites might mediate the association between pBMI and GDM risk. The findings may help to elucidate mechanistic pathways and identify early biomarkers for further preventive and therapeutic targets.

## 2. Methods

### 2.1. Study Population

Pregnant women who routinely underwent obstetric examination and delivery in Shanghai Sixth People’s Hospital from 2017 to 2020 were enrolled. Blood samples were collected at 10–20 weeks’ gestation for prenatal genetic screening (mean: 16.8 weeks), and women who provided blood samples were included in this study. After exclusion of the women with known pregestational diabetes, use of antidiabetic drugs, and a history of bariatric surgery, we included 100 pregnant women with GDM as the case group and 100 pregnant women without GDM matched by age as the control group. Information on demographic characteristics, reproductive history, and laboratory examination was extracted using the electronic medical record. The study was approved by the ethics committee of Shanghai Sixth People’s Hospital and was conducted in accordance with the principles of the Declaration of Helsinki.

The validation population were obtained from the University of Hong Kong‐Shenzhen Hospital in 2015–2018, comprising 200 healthy pregnant women and 200 GDM women, from whom blood samples were collected during oral glucose tolerance test (OGTT) in 24–27 weeks’ gestation. The details of the population have been published previously [14, 15]. This population was used to validate the effect of identified metabolites and pathways on the occurrence of GDM. The study was approved by the ethics committee of the University of Hong Kong‐Shenzhen Hospital.

#### 2.1.1. Assessment of GDM and pBMI

The assessment for GDM women was based on a standard 75 g 2 h OGTT at 24–28 weeks of gestation according to the International Association of the Diabetes and Pregnancy Study Group. GDM was defined if any of the following criteria were met: fasting plasma glucose (FPG) ≥ 5.1 mmol/L, 1 h plasma glucose ≥ 10.0 mmol/L, or 2 h plasma glucose ≥ 8.5 mmol/L. Women with all values below the thresholds were considered normal.

Maternal height and weight were measured at each obstetric examination visit, while pregestational weight was also collected by self‐reported at the first visit. Body mass index (BMI) was calculated as body weight (in kilogram)/height squared (in square meter).

#### 2.1.2. Metabolomic Measurement

Frozen serum samples were stored at −80°C until analysis. Targeted metabolomic assays measured by ultra performance liquid chromatography–tandem mass spectrometry (UPLC‐MS/MS) and processed by MassLynx software. One hundred and sixty‐five metabolomics were detected, mainly encompassing fatty acids, amino acids, organic acids, carnitines/acylcarnitines, bile acids, carbohydrates, and short‐chain fatty acids (SCFAs). The limit of detection was applied to fill in missing values of quantitative metabolomic data.

#### 2.1.3. Statistical Analyses

Baseline characteristics of the study population were presented as means ± standard deviations (SDs) for continuous variables or numbers (percentages) for categorical variables, tested by t‐test and chi‐square test as appropriate.

All metabolites were scaled to a standard *z*‐score with a mean of zero and an SD of one for statistical analyses. Multivariable linear models were performed to examine the association between pBMI and serum metabolites, and multivariable logistic models were performed to examine the association between GDM and serum metabolites. Both multivariable models were adjusted for age and parity. We conducted pathway enrichment analyses among significantly associated metabolites using MetaboAnalyst 6.0 (https://www.metaboanalyst.ca/) based on the Kyoto Encyclopedia of Genes and Genomes (KEGG) database.

We used two strategies in this study to analyze the mediating role of serum metabolites between pBMI and GDM risk. First, we employed a causal mediation model proposed by VanderWeele for each shared serum metabolite to examine the independent mediating effect of each individual metabolite. Second, considering the interaction and joint effect of metabolites, we performed hierarchical clustering on the shared metabolites and generated LVs to represent the variance of correlated metabolites within each cluster using the ClustVarLV package. Posterior enrichment analyses for metabolite clusters within each LV were then used to identify the pathway of metabolite sets. The LVs were used to investigate their mediating role in the relationship between pBMI and GDM. Serial mediation analysis was used to evaluate the potential path of multiple LVs. To validate the effect of the identified pathway of LVs, we conducted a mediation analysis on the external population with blood samples collected during the OGTT. The causal mediation model was conducted by the mediation package, and the serial mediation model was conducted by the bruceR package.

All statistical analyses were performed using R software (Version 4.4.2). *p* value less than 0.05 was considered statistically significant.

## 3. Results

### 3.1. Characteristics of Study Participants

The characteristics of study participants are shown in Table 1. The mean age of the 200 pregnant women was 29.12 (SD 3.22) years, and the mean pBMI was 21.62 (SD 3.01) kg/m^2^. Compared with women without GDM, women with GDM were more likely to have higher pBMI, parity, evaluated fasting glucose, and HbA1c levels at early pregnancy, as well as OGTT results including fasting, 1 and 2 h. There was no difference in age, gravidity, and education.

**Table 1:** Characteristics of study participants by GDM.

**Characteristic**	**Overall** **N** = 200	**Non-GDM** **N** = 100	**GDM** **N** = 100	**p** **value**
Age, years	29.12 (3.22)	29.12 (3.22)	29.12 (3.22)	> 0.9
pBMI (kg/m^2^)	21.62 (3.01)	21.00 (2.78)	22.23 (3.12)	0.004
Gravidity				0.265
1–2	146 (73.00%)	69 (69.00%)	77 (77.00%)	
≥ 3	54 (27.00%)	31 (31.00%)	23 (23.00%)	
Parity				0.033
0	90 (45.00%)	53 (53.00%)	37 (37.00%)	
≥ 1	110 (55.00%)	47 (47.00%)	63 (63.00%)	
Education				0.317
College	153 (76.50%)	73 (73.00%)	80 (80.00%)	
Other	47 (23.50%)	27 (27.00%)	20 (20.00%)	
FPG, mmol/L	4.79 (0.34)	4.73 (0.31)	4.85 (0.35)	0.008
HbA1c, %	5.21 (0.25)	5.16 (0.22)	5.27 (0.28)	0.004
OGTT‐FPG, mmol/L	4.71 (0.45)	4.53 (0.27)	4.90 (0.51)	< 0.001
OGTT‐1 h, mmol/L	8.39 (1.73)	7.36 (1.24)	9.41 (1.54)	< 0.001
OGTT‐2 h, mmol/L	6.94 (1.53)	6.07 (1.02)	7.82 (1.46)	< 0.001

Abbreviations: FPG, fasting plasma glucose; GDM, gestational diabetes mellitus; HbA1c, glycated hemoglobin; OGTT, oral glucose tolerance test; pBMI, prepregnancy body mass index.

### 3.2. Associations Among pBMI, Serum Metabolites, and GDM

The association between pBMI and GDM is presented in Figure 1a. It was shown that pBMI was positively associated with an increased risk of GDM in either the crude or multivariate‐adjusted models, with ORs of 1.15 (95% CI: 1.05–1.28) and 1.18 (95% CI: 1.07–1.31), respectively.

Figure 1Associations among pBMI, serum metabolites, and GDM. (a) The association between pBMI and GDM risk. (b) The association between pBMI and serum metabolites. (c) The association between serum metabolites and GDM risk. All regression models were adjusted for age and parity.

**Figure:**
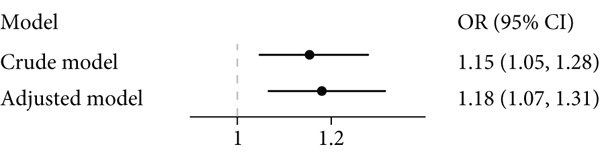
(a)

**Figure:**
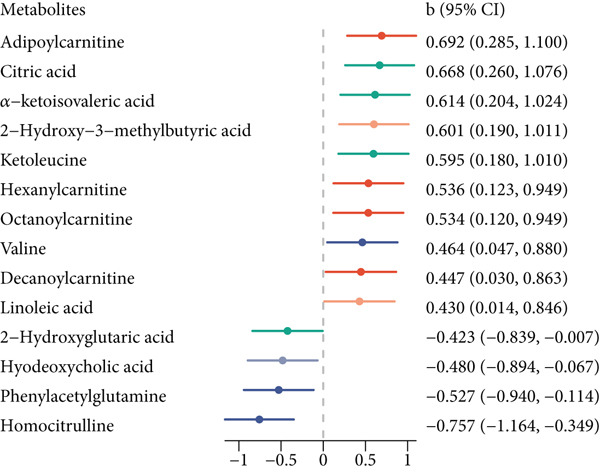
(b)

**Figure:**
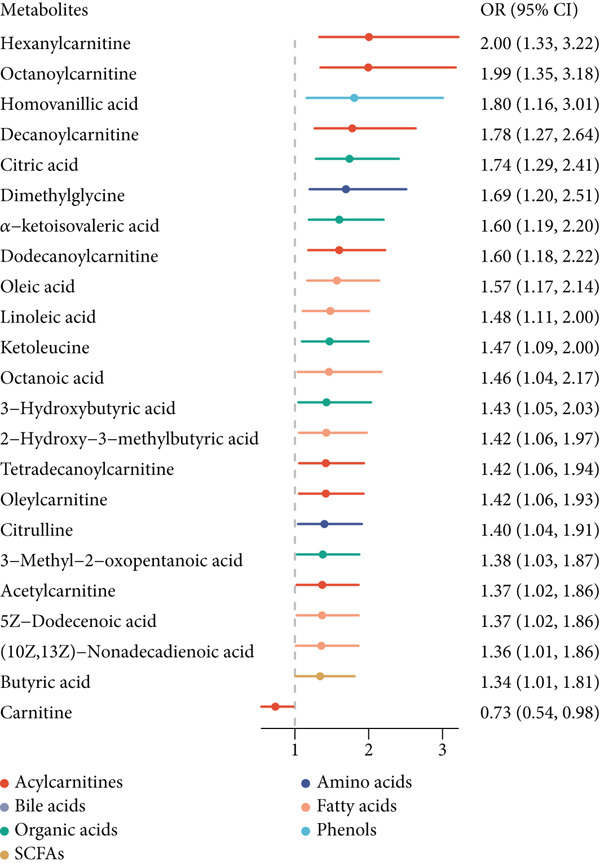
(c)

A total of 14 metabolites were found to be significantly associated with pBMI (Table S1 and Figure 1b). Among them, 10 metabolites were positively associated with pBMI, while 4 metabolites were negatively associated with pBMI. Pathway enrichment analysis suggested that these pBMI‐related metabolites were enriched in several metabolic pathways (Figure 2b). Four metabolic pathways reached statistical significance at *p* < 0.05, and valine, leucine, and isoleucine biosynthesis; valine, leucine, and isoleucine degradation; and pantothenate and CoA biosynthesis also achieved an FDR < 0.05.

Figure 2The shared metabolites and pathway between pBMI and GDM. (a) Venn diagram of pBMI‐related and GDM‐related metabolites. (b) Pathway enrichment analysis of pBMI‐related and GDM‐related metabolites.

**Figure:**
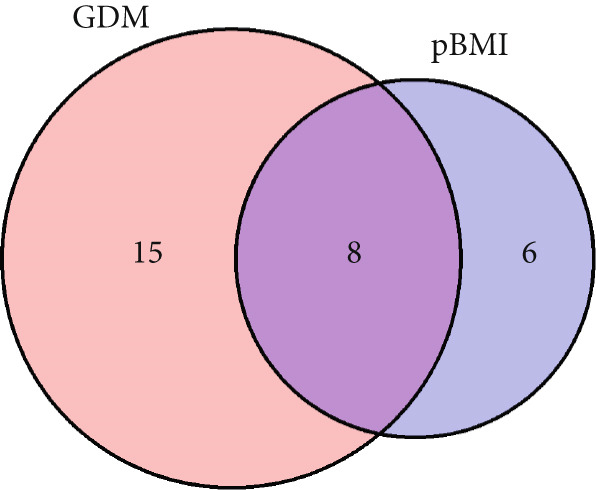
(a)

**Figure:**
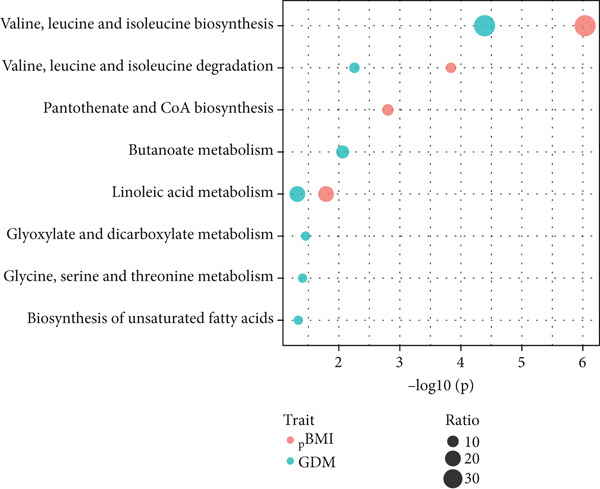
(b)

There were 23 metabolites exhibiting significant associations with the risk of GDM (Table S2 and Figure 1c). Twenty‐two metabolites were positively associated with GDM, and only carnitine was negatively associated with GDM. Several metabolic pathways were enriched for GDM‐related metabolites, among which seven metabolic pathways reached statistical significance at *p* < 0.05, and valine, leucine, and isoleucine biosynthesis also achieved an FDR < 0.05 (Figure 2b).

Combining the pBMI‐related metabolites and the GDM‐related metabolites, there are eight overlapping metabolites (Figure 2a). The shared metabolites consisted of three organic acids, three acylcarnitines, and two fatty acids. The shared dysregulated metabolic pathways for pBMI and GDM mainly included amino acid metabolism and fatty acid metabolism, such as valine, leucine, and isoleucine biosynthesis; valine, leucine, and isoleucine degradation; and linoleic acid metabolism. Indeed, the dysregulated metabolic pathways were predominated by valine, leucine, and isoleucine biosynthesis for both pBMI and GDM (Figure 2b).

**Figure 3:**
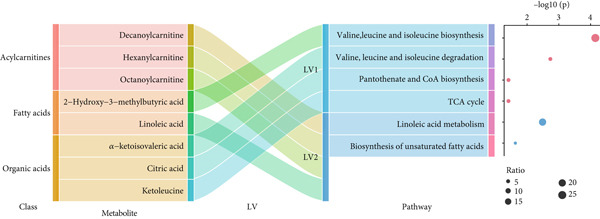
Clustered latent variable of shared metabolites for pBMI and GDM. LV, latent variable.

Eight shared metabolites were then clustered into two LVs by hierarchical clustering (Figure 3). LV1 included citric acid, alpha‐ketoisovaleric acid, ketoleucine, and 2‐hydroxy‐3‐methylbutyric acid, mainly enriched in valine, leucine, and isoleucine biosynthesis. LV2 included octanoylcarnitine, decanoylcarnitine, hexanoylcarnitine, and linoleic acid, mainly enriched in linoleic acid metabolism.

### 3.3. Mediating Role of Metabolites in the Association Between pBMI and Risk of GDM

We further conducted mediation analysis to evaluate whether shared metabolites were involved in the association between pBMI and risk of GDM. Among eight shared metabolites, five individual metabolites played a significant mediating role between pBMI and risk of GDM (Figure 4a and Table S3). The indirect effects of citric acid, octanoylcarnitine, hexanoylcarnitine, alpha‐ketoisovaleric acid, and decanoylcarnitine were 0.0014 (95% CI: 0.0001–0.0057), 0.0013 (95% CI: 0.0001–0.0053), 0.0013 (95% CI: 0.0001–0.0054), 0.0011 (95% CI: 0.0000–0.0046), and 0.0010 (95% CI: 0.0000–0.0043), accounting for 19.6%, 19.6%, 19.5%, 15.1%, and 14.1% of the total effect, respectively.

Figure 4The mediating role of serum metabolites in early‐mid pregnancy in the association between pBMI and GDM risk. (a) The mediating role of individual metabolites in the association between pBMI and GDM risk. (b) The mediating role of LVs of metabolites in the association between pBMI and GDM risk. All mediation models were adjusted for age and parity. IE, indirect effect; Prop, mediated proportion; LV, latent variable.

**Figure:**
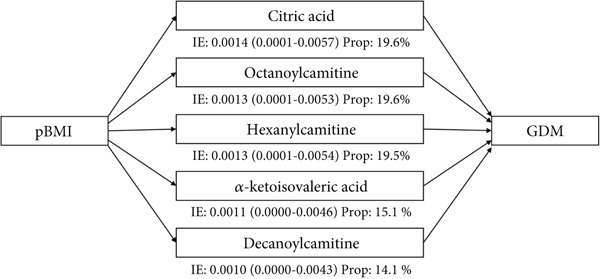
(a)

**Figure:**
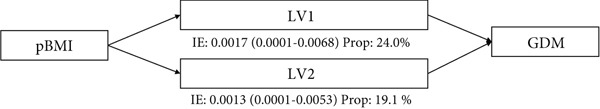
(b)

To explore the potential coeffect of metabolites underlying this process, we constructed a mediation analysis for two LVs of shared metabolites. Both LVs presented significant mediation effects between pBMI and GDM risk (Figure 4b and Table S3). LV1 showed an indirect effect of 0.0017 (95% CI: 0.0001–0.0068) and a mediated proportion of 24.0%. LV2 showed an indirect effect of 0.0013 (95% CI: 0.0001–0.0053) and a mediated proportion of 19.1%. Serial mediation analyses were performed to evaluate the potential path of LV1 and LV2 between pBMI and GDM risk. Table S4 shows that the serial mediated effect of the LV1–LV2 path was significant, with a proportion of mediation of 5.3%, and the LV2–LV1 path was nonsignificant on the association of pBMI with GDM risk, indicating LV1 with more important effect.

### 3.4. Validating the Effect of Identified Metabolic Pathway

To validate the effect of valine, leucine, and isoleucine biosynthesis in the association between pBMI and risk of GDM, we evaluated the roles of valine, leucine, and isoleucine, categorized as branched‐chain amino acids (BCAAs), on the period of the OGTT. pBMI and GDM showed significantly positive associations with total BCAAs and each of them during the OGTT period (Table 2). In the mediation analysis of BCAAs, total BCAAs and valine played significant mediating roles in the relationship between pBMI and GDM, accounting for 23.0% and 13.2% of the total effect, respectively.

**Table 2:** The mediating role of BCAAs during the OGTT period in the association between pBMI and GDM risk.

	**pBMI and BCAAs**		**BCAAs and GDM**		**Indirect effect of BCAAs**		
**Mediator**	**b** **(95% CI)**	**p**	**OR (95% CI)**	**p**	**Indirect effect (95% CI)**	**p**	**Prop (%)**
BCAAs	0.460 (0.201–0.719)	0.001	1.35 (1.10–1.67)	0.004	0.0028 (0.0004 to 0.0071)	0.006	23.0
Valine	0.301 (0.039–0.563)	0.025	1.31 (1.07–1.61)	0.010	0.0016 (0.0000 to 0.0049)	0.040	13.2
Leucine	0.435 (0.176–0.695)	0.001	1.24 (1.02–1.52)	0.036	0.0018 (−0.0001 to 0.0054)	0.068	14.7
Isoleucine	0.350 (0.090–0.611)	0.009	1.25 (1.02–1.56)	0.033	0.0016 (−0.0000 to 0.0046)	0.056	12.2

*Note:* All models were adjusted for age and parity.

Abbreviations: BCAAs, branched‐chain amino acids; Prop, mediated proportion.

## 4. Discussion

In this study, we found 14 and 23 metabolites in early‐mid pregnancy significantly associated with pBMI and GDM risk, respectively, with 8 metabolites overlapping. The pathway analyses identified valine, leucine, and isoleucine biosynthesis as the top and shared pathway enriched for pBMI and GDM. The association of pBMI on GDM risk was significantly mediated by metabolites, including five individual metabolites and two clustered LVs. The LV, enriched in valine, leucine, and isoleucine biosynthesis, played the most significant mediating effect and was further validated during the OGTT period.

Accumulating evidence has revealed metabolite change during pregnancy for pBMI and GDM, despite the results not being consistent, possibly due to heterogeneous study designs, limited sample size, ethnic diversity, and variations in gestational stage [7–10, 16]. While most metabolomic research has separately examined pBMI and GDM, identifying either the same or different metabolites, few studies have systematically explored their metabolic interrelationships. Hyperglycaemia and Adverse Pregnancy Outcome (HAPO) study identified both shared and unique associations of amino acids and acylcarnitines with BMI and glucose at approximately 28 weeks’ gestation [17, 18]. A longitudinal metabolic profiling study found metabolic alterations related with GDM resembled the alterations related with obesity [12]. Some other studies have found the impact of obesity on GDM‐related metabolites. Our study elucidates the critical role of early‐to‐mid pregnancy metabolites in the pBMI‐GDM association, finding eight metabolites simultaneously significantly associated with both pBMI and GDM. These eight metabolites consisting of three organic acids, three acylcarnitines, and two fatty acids were all showed positive associations for pBMI and GDM, which was similar to the previous studies [19]. Among eight metabolites, five individual metabolites and two derived LVs participated in mediating the association between pBMI and GDM. While the biological pathways linking adiposity to glucose dysregulation remain incompletely understood, metabolomic profiling enables the identification of intermediate biomarkers and metabolic pathways that may explain how pBMI influences GDM pathogenesis. Our research provides novel temporal insights into how pBMI‐driven metabolic perturbations may predispose to subsequent GDM development, which contribute to further preventive and therapeutic targets.

Two of the three overlapping organic acids, alpha‐ketoisovaleric acid and ketoleucine, as well as one fatty acid, 2‐hydroxy‐3‐methylbutyric acid, are derivatives of BCAAs. Intriguingly, we have also pointed out valine, leucine, and isoleucine biosynthesis as the key pathway linking pBMI and GDM risk. These three amino acids, which belong to the BCAA group, are essential for human nutrition. Consistent reports have proved the positive association between BCAAs and obesity and diabetes in the general population, establishing them as reliable biomarkers for obesity‐associated insulin resistance [20, 21]. Findings in pregnant women have been inconsistent during different stages of pregnancy. A recent study found that BCAA concentrations at mid pregnancy, but not at early pregnancy, were positively associated with GDM risk, which was consistent with our study [22]. Although valine was significantly associated with pBMI but not GDM during early‐mid pregnancy, metabolites within BCAA‐related metabolic pathways, such as alpha‐ketoisovaleric acid (a valine derivative) and ketoleucine (a leucine derivative), showed significant associations with both pBMI and GDM. Notably, this relationship extended to BCAA levels themselves during mid‐late pregnancy, suggesting a progressive metabolic dysregulation underlying the pathogenesis of the association between pBMI and GDM.

Some mechanisms possibly supported the mediating role of BCAAs and their derivatives in the association between pBMI and GDM risk [23]. On the one hand, obesity affects the activity of key enzymes involved in BCAA catabolism. Animal experiments have shown that obese rats performed decreased BCKDH complex enzymatic activity and increased phosphorylation of the E1 component of the complex in adipose tissue, leading to an evaluation in the concentration of BCAAs and their derivatives [24]. On the other hand, BCAAs and their derivatives may disrupt glucose metabolism through improving insulin resistance. High levels of BCAAs activate mammalian target of rapamycin complex 1 (mTORC1), resulting in insulin resistance through the phosphorylation of insulin receptor substrate 1 (IRS‐1) [25, 26]. Besides, as incompletely metabolized products of BCAAs, the evaluated alpha‐ketoisovaleric acid and ketoleucine exhibit significant neurotoxicity and metabolic toxicity. Accumulation of toxic BCAA metabolites may be induction of oxidative stress, mitochondrial dysfunction, impaired insulin action, and ultimately to perturbation of glucose homeostasis [27, 28].

Citric acid is an essential intermediate of the TCA cycle. We found that citric acid itself, as well as the metabolite set composed of it and BCAA derivatives, both mediate the pBMI‐GDM association. Cluster analysis reveals a correlation of citric acid and BCAA derivatives, indicating the complex disorders of glucose metabolism and BCAA metabolism interaction associated with pBMI and GDM [28, 29].

We also found that acylcarnitines may contribute to the relationship between pBMI and GDM risk. Acylcarnitines exhibited the strongest positive associations among all metabolites in the study, and medium‐chain acylcarnitines, including hexanoylcarnitine (C6), octanoylcarnitine (C8), and decanoylcarnitine (C10), mediated the effects of pBMI on the increased risk of GDM. These results were consistent with previous studies that medium‐chain acylcarnitines were elevated in GDM and overweight women [30–32]. Elevated levels of acylcarnitines may indicate impaired *β*‐oxidation of fatty acids and altered mitochondrial metabolism induced by pBMI [33]. Cluster analysis and pathway analysis indicated the coeffect between medium‐chain acylcarnitines and linoleic acid linking pBMI and GDM risk. However, further biological studies are necessary to fully understand the specific mechanism of medium‐chain acylcarnitines.

To the best of our knowledge, this is the first study to characterize the shared metabolic profile of pBMI and GDM in early‐mid pregnancy. We elucidate the role of metabolites for subsequent GDM risk induced by pBMI based on a temporal design. However, several limitations of this study were supposed to be considered. First, our study was deficient in information about family history, healthy lifestyle, and dietary habits that should be adjusted in the multivariable analyses. Second, pBMI was collected at the first visit in the obstetric examination, which may cause recall bias. But there was a 95.4% correlation of pBMI with BMI measured at the first visit, indicating its reliability. Finally, a single assessment for serum metabolites may not detect long‐term exposure or reflect dynamic changes, so repeated measurements in the same population are recommended for future studies.

## 5. Conclusion

In this study, we found eight shared metabolites in early‐mid pregnancy significantly associated with both pBMI and GDM risk, including organic acids, acylcarnitines, and fatty acids. Five individual metabolites and two clustered LVs might be the mediators in the association between pBMI and GDM risk, highlighting the significant role of valine, leucine, and isoleucine biosynthesis. The study contributed to the underlying mechanism that connects pBMI to the risk of GDM and provided insights into the development of potential therapeutic opportunities.

## Disclosure

All authors reviewed the manuscript.

## Conflicts of Interest

The authors declare no conflicts of interest.

## Author Contributions

Suna Wang and Yanwei Zheng contributed equally to this work. Xiangtian Yu and Rongzhen Jiang were responsible for the conception and design of the study. Suna Wang and Yanwei Zheng were involved in data collection, data analysis, and drafting the manuscript. Mingjuan Luo, Jingyi Guo and Wei Chen were involved in data collection and interpretation.

## Funding

This study was supported by the National Key Research and Development Program of China (10.13039/501100012166, 2022YFA1004800), Fundamental Research Funds for the Central Universities (10.13039/501100012226, YG2023QNB20), the Clinical Research Plan of SHDC (SHDC2022CRS044 and SHDC2023CRS029), and the Shanghai Sixth People’s Hospital Affiliated to Shanghai Jiao Tong University School of Medicine Clinical Research (hnhg202126).

## Supporting information


**Supporting Information** Additional supporting information can be found online in the Supporting Information section. Table S1 The association between pBMI and serum metabolites. Table S2 The association between serum metabolites and GDM risk. Table S3 The mediating role of individual and LV metabolites in early‐mid pregnancy in the association between pBMI and GDM risk. Table S4 The serial mediated effect of LVs in the association between pBMI and GDM risk.

## Data Availability

The data that support the findings of this study are available from the corresponding authors upon reasonable request.
