# Supplementary material for: Metabolites in Early‐Mid Pregnancy Mediate the Association Between Prepregnancy Body Mass Index and Risk of Gestational Diabetes Mellitus
Source: J Diabetes Res. 2026 Feb 4;2026:6303241. doi: 10.1155/jdr/6303241 (PMC12872595; doi:10.1155/jdr/6303241)
Supplement: Supplementary file 1 — Supporting Information Additional supporting information can be found online in the Supporting Information section. Table S1 The association between pBMI and serum metabolites. Table S2 The association between serum metabolites and GDM risk. Table S3 The mediating role of individual and LV metabolites in early‐mid pregnancy in the association between pBMI and GDM risk. Table S4 The serial mediated effect of LVs in the association between pBMI and GDM risk. [file JDR-2026-6303241-s001.docx]

**Supplementary materials**

Table S1 The association between pBMI and serum metabolites.

Table S2 The association between serum metabolites and GDM risk.

Table S3 The mediating role of individual and LVs metabolites in early-mid pregnancy in the association between pBMI and GDM risk

Table S4 The serial mediated effect of LVs in the association between pBMI and GDM risk

Table S1 The association between pBMI and serum metabolites.

| Metabolite | Class | b (95% CI) | P value |
| --- | --- | --- | --- |
| Acetylglycine | Amino Acids | 0.136 (-0.285, 0.556) | 0.525 |
| Phenylacetylglutamine | Amino Acids | -0.527 (-0.940, -0.114) | 0.013 |
| Aminocaproic acid | Amino Acids | -0.101 (-0.519, 0.318) | 0.636 |
| Citrulline | Amino Acids | -0.059 (-0.485, 0.368) | 0.787 |
| Creatine | Amino Acids | -0.345 (-0.763, 0.073) | 0.105 |
| Dimethylglycine | Amino Acids | 0.395 (-0.022, 0.812) | 0.064 |
| GABA | Amino Acids | -0.050 (-0.470, 0.369) | 0.814 |
| Glycine | Amino Acids | 0.003 (-0.418, 0.423) | 0.990 |
| Alanine | Amino Acids | 0.193 (-0.229, 0.615) | 0.368 |
| alpha-Aminobutyric acid | Amino Acids | 0.004 (-0.416, 0.424) | 0.985 |
| Arginine | Amino Acids | 0.231 (-0.189, 0.652) | 0.280 |
| Asparagine | Amino Acids | -0.308 (-0.724, 0.108) | 0.146 |
| Aspartic acid | Amino Acids | 0.258 (-0.168, 0.683) | 0.235 |
| Glutamic acid | Amino Acids | 0.257 (-0.160, 0.674) | 0.226 |
| Glutamine | Amino Acids | -0.111 (-0.536, 0.313) | 0.605 |
| Histidine | Amino Acids | -0.055 (-0.475, 0.365) | 0.796 |
| Homocitrulline | Amino Acids | -0.757 (-1.164, -0.349) | 3.18e-04 |
| Isoleucine | Amino Acids | 0.260 (-0.158, 0.678) | 0.221 |
| Leucine | Amino Acids | 0.387 (-0.032, 0.806) | 0.070 |
| Lysine | Amino Acids | -0.165 (-0.589, 0.259) | 0.443 |
| Methionine | Amino Acids | -0.109 (-0.531, 0.312) | 0.610 |
| Pipecolic acid | Amino Acids | 0.125 (-0.294, 0.544) | 0.558 |
| Proline | Amino Acids | 0.384 (-0.031, 0.799) | 0.069 |
| Serine | Amino Acids | -0.061 (-0.483, 0.361) | 0.777 |
| Threonine | Amino Acids | 0.320 (-0.106, 0.746) | 0.141 |
| Tryptophan | Amino Acids | -0.218 (-0.639, 0.203) | 0.308 |
| Tyrosine | Amino Acids | -0.048 (-0.467, 0.372) | 0.823 |
| Valine | Amino Acids | 0.464 (0.047, 0.880) | 0.029 |
| Methylcysteine | Amino Acids | -0.197 (-0.615, 0.221) | 0.354 |
| N-Acetylaspartic acid | Amino Acids | -0.113 (-0.533, 0.307) | 0.597 |
| N-Acetylglutamine | Amino Acids | 0.130 (-0.290, 0.550) | 0.542 |
| N-Acetylserine | Amino Acids | -0.208 (-0.626, 0.210) | 0.327 |
| Ornithine | Amino Acids | 0.398 (-0.024, 0.820) | 0.065 |
| Benzenebutanoic acid | Benzenoids | -0.148 (-0.566, 0.270) | 0.486 |
| Phenylpyruvic acid | Benzenoids | -0.389 (-0.805, 0.026) | 0.066 |
| Hippuric acid | Benzoic Acids | 0.105 (-0.320, 0.531) | 0.625 |
| Beta_Ursodeoxycholic Acid | Bile Acids | -0.009 (-0.428, 0.410) | 0.967 |
| Chenodeoxycholic acid | Bile Acids | 0.367 (-0.048, 0.783) | 0.083 |
| Cholic acid | Bile Acids | 0.342 (-0.076, 0.759) | 0.108 |
| Deoxycholic acid | Bile Acids | 0.314 (-0.104, 0.733) | 0.140 |
| Glycochenodeoxycholic acid | Bile Acids | -0.084 (-0.503, 0.334) | 0.691 |
| Glycocholic acid | Bile Acids | 0.095 (-0.324, 0.513) | 0.656 |
| Glycodeoxycholic acid | Bile Acids | -0.147 (-0.566, 0.272) | 0.491 |
| Glycohyocholic acid | Bile Acids | 0.122 (-0.302, 0.545) | 0.572 |
| Glycolithocholic acid | Bile Acids | -0.328 (-0.745, 0.088) | 0.122 |
| Glycoursodeoxycholic acid | Bile Acids | -0.406 (-0.822, 0.011) | 0.056 |
| Hyodeoxycholic acid | Bile Acids | -0.480 (-0.894, -0.067) | 0.023 |
| isolithocholic acid | Bile Acids | -0.034 (-0.453, 0.385) | 0.874 |
| Lithocholic acid | Bile Acids | -0.380 (-0.797, 0.036) | 0.073 |
| Taurochenodeoxycholic acid | Bile Acids | -0.090 (-0.511, 0.332) | 0.675 |
| Taurocholic acid | Bile Acids | 0.069 (-0.350, 0.489) | 0.745 |
| Taurodeoxycholic acid | Bile Acids | -0.249 (-0.667, 0.169) | 0.241 |
| Ursodeoxycholic acid | Bile Acids | -0.082 (-0.504, 0.340) | 0.702 |
| Fructose | Carbohydrates | -0.196 (-0.614, 0.222) | 0.356 |
| Gluconolactone | Carbohydrates | 0.337 (-0.079, 0.752) | 0.112 |
| Glucose | Carbohydrates | 0.284 (-0.135, 0.703) | 0.183 |
| Maltose | Carbohydrates | -0.346 (-0.762, 0.069) | 0.102 |
| Xylose | Carbohydrates | -0.021 (-0.442, 0.401) | 0.923 |
| Erythronic acid | Carbohydrates | -0.196 (-0.617, 0.226) | 0.361 |
| Galactonic acid | Carbohydrates | 0.210 (-0.208, 0.629) | 0.323 |
| Glyceric acid | Carbohydrates | -0.075 (-0.498, 0.347) | 0.725 |
| Lactulose | Carbohydrates | -0.193 (-0.612, 0.227) | 0.366 |
| N-Acetylglucosamine | Carbohydrates | 0.009 (-0.409, 0.428) | 0.965 |
| N-Acetylneuraminic acid | Carbohydrates | -0.296 (-0.717, 0.125) | 0.167 |
| Tartaric acid | Carbohydrates | -0.060 (-0.480, 0.359) | 0.777 |
| Adipoylcarnitine | Carnitines | 0.692 (0.285, 1.100) | 9.71e-04 |
| Carnitine | Carnitines | -0.342 (-0.758, 0.074) | 0.107 |
| Decanoylcarnitine | Carnitines | 0.447 (0.030, 0.863) | 0.036 |
| Glutarylcarnitine | Carnitines | 0.098 (-0.325, 0.520) | 0.649 |
| Hexanylcarnitine | Carnitines | 0.536 (0.123, 0.949) | 0.011 |
| Isovalerylcarnitine | Carnitines | 0.214 (-0.204, 0.633) | 0.314 |
| Acetylcarnitine | Carnitines | 0.089 (-0.333, 0.510) | 0.679 |
| Dodecanoylcarnitine | Carnitines | 0.318 (-0.104, 0.740) | 0.139 |
| Linoleylcarnitine | Carnitines | 0.347 (-0.076, 0.769) | 0.107 |
| Malonylcarnitine | Carnitines | 0.285 (-0.135, 0.705) | 0.182 |
| Methylmalonylcarnitine | Carnitines | 0.011 (-0.417, 0.439) | 0.961 |
| Tetradecanoylcarnitine | Carnitines | 0.255 (-0.169, 0.679) | 0.237 |
| Octanoylcarnitine | Carnitines | 0.534 (0.120, 0.949) | 0.012 |
| Oleylcarnitine | Carnitines | 0.400 (-0.025, 0.824) | 0.065 |
| Palmitoylcarnitine | Carnitines | 0.264 (-0.160, 0.689) | 0.221 |
| Propionylcarnitine | Carnitines | -0.254 (-0.670, 0.163) | 0.231 |
| Stearylcarnitine | Carnitines | -0.207 (-0.627, 0.214) | 0.333 |
| 2-Methylbutyroylcarnitine | Carnitines | 0.198 (-0.222, 0.618) | 0.353 |
| 3-Hydroxylisovalerylcarnitine | Carnitines | -0.167 (-0.596, 0.261) | 0.442 |
| Adipic acid | Fatty Acids | -0.189 (-0.607, 0.229) | 0.373 |
| Adrenic acid | Fatty Acids | -0.008 (-0.426, 0.411) | 0.971 |
| alpha-Linolenic acid | Fatty Acids | 0.332 (-0.087, 0.751) | 0.120 |
| Arachidonic acid | Fatty Acids | 0.078 (-0.340, 0.497) | 0.713 |
| Azelaic acid | Fatty Acids | 0.048 (-0.374, 0.470) | 0.823 |
| 2-Butenoic acid | Fatty Acids | 0.247 (-0.171, 0.664) | 0.245 |
| Citraconic acid | Fatty Acids | 0.053 (-0.371, 0.477) | 0.806 |
| Citramalic acid | Fatty Acids | -0.019 (-0.437, 0.400) | 0.930 |
| Decanoic acid | Fatty Acids | -0.089 (-0.515, 0.337) | 0.681 |
| DHA | Fatty Acids | -0.125 (-0.549, 0.300) | 0.563 |
| DPAn-6 | Fatty Acids | -0.076 (-0.495, 0.343) | 0.720 |
| DPA | Fatty Acids | 0.250 (-0.168, 0.668) | 0.239 |
| Dodecanoic acid | Fatty Acids | -0.245 (-0.677, 0.187) | 0.265 |
| EPA | Fatty Acids | -0.194 (-0.616, 0.228) | 0.366 |
| gamma-Linolenic acid | Fatty Acids | 0.409 (-0.007, 0.825) | 0.054 |
| Heptanoic acid | Fatty Acids | 0.115 (-0.304, 0.534) | 0.590 |
| Itaconic acid | Fatty Acids | -0.003 (-0.421, 0.416) | 0.991 |
| Linoleic acid | Fatty Acids | 0.430 (0.014, 0.846) | 0.043 |
| Methylglutaric acid | Fatty Acids | -0.081 (-0.500, 0.337) | 0.703 |
| 9E-tetradecenoic acid | Fatty Acids | 0.179 (-0.240, 0.597) | 0.401 |
| Myristic acid | Fatty Acids | 0.093 (-0.328, 0.514) | 0.663 |
| Myristoleic acid | Fatty Acids | -0.064 (-0.494, 0.365) | 0.768 |
| Nonanoic acid | Fatty Acids | -0.354 (-0.772, 0.063) | 0.095 |
| Octanoic acid | Fatty Acids | 0.056 (-0.366, 0.478) | 0.795 |
| Oleic acid | Fatty Acids | 0.350 (-0.071, 0.770) | 0.103 |
| Palmitoleic acid | Fatty Acids | 0.119 (-0.306, 0.544) | 0.581 |
| Pentadecanoic acid | Fatty Acids | 0.033 (-0.386, 0.453) | 0.875 |
| Pimelic acid | Fatty Acids | -0.251 (-0.671, 0.169) | 0.240 |
| Ricinoleic acid | Fatty Acids | -0.145 (-0.562, 0.273) | 0.496 |
| Sebacic acid | Fatty Acids | -0.126 (-0.544, 0.291) | 0.551 |
| Tridecanoic acid | Fatty Acids | 0.027 (-0.393, 0.447) | 0.900 |
| Undecanoic acid | Fatty Acids | -0.103 (-0.524, 0.318) | 0.631 |
| (10Z,13Z)-Nonadecadienoic acid | Fatty Acids | 0.257 (-0.162, 0.676) | 0.228 |
| 10Z-Heptadecenoic acid | Fatty Acids | 0.250 (-0.177, 0.676) | 0.249 |
| 2-Hydroxy-3-methylbutyric acid | Fatty Acids | 0.601 (0.190, 1.011) | 0.004 |
| 3-Hydroxyisovaleric acid | Fatty Acids | -0.244 (-0.663, 0.176) | 0.254 |
| 3-Methyladipic acid | Fatty Acids | -0.011 (-0.436, 0.413) | 0.959 |
| 5Z-Dodecenoic acid | Fatty Acids | 0.137 (-0.283, 0.558) | 0.521 |
| Dihomo-gamma-linolenic acid | Fatty Acids | 0.317 (-0.099, 0.733) | 0.134 |
| 9-Pentadecenoic acid | Fatty Acids | -0.027 (-0.446, 0.391) | 0.897 |
| Indoleacetic acid | Indoles | -0.128 (-0.548, 0.293) | 0.549 |
| Indole-3-propionic acid | Indoles | -0.212 (-0.630, 0.205) | 0.318 |
| AMP | Nucleotides | -0.243 (-0.670, 0.185) | 0.265 |
| alpha-Hydroxyisobutyric acid | Organic Acids | -0.163 (-0.580, 0.255) | 0.444 |
| alpha-Ketoisovaleric acid | Organic Acids | 0.614 (0.204, 1.024) | 0.004 |
| Aconitic acid | Organic Acids | 0.241 (-0.176, 0.658) | 0.256 |
| Citric acid | Organic Acids | 0.668 (0.260, 1.076) | 0.001 |
| Fumaric acid | Organic Acids | -0.202 (-0.620, 0.215) | 0.340 |
| Glutaconic acid | Organic Acids | 0.050 (-0.371, 0.471) | 0.816 |
| Glutaric acid | Organic Acids | -0.320 (-0.736, 0.097) | 0.132 |
| Glycolic acid | Organic Acids | -0.079 (-0.501, 0.343) | 0.713 |
| Guanidoacetic acid | Organic Acids | -0.152 (-0.571, 0.267) | 0.475 |
| Isocitric acid | Organic Acids | 0.285 (-0.135, 0.706) | 0.182 |
| Ketoleucine | Organic Acids | 0.595 (0.180, 1.010) | 0.005 |
| Lactic acid | Organic Acids | 0.068 (-0.352, 0.488) | 0.750 |
| Malic acid | Organic Acids | -0.116 (-0.536, 0.304) | 0.587 |
| Maleic acid | Organic Acids | 0.120 (-0.299, 0.539) | 0.573 |
| Methylmalonic acid | Organic Acids | -0.376 (-0.798, 0.047) | 0.081 |
| Oxoadipic acid | Organic Acids | -0.033 (-0.453, 0.387) | 0.877 |
| Oxoglutaric acid | Organic Acids | 0.414 (-0.003, 0.832) | 0.052 |
| Pyruvic acid | Organic Acids | 0.080 (-0.342, 0.502) | 0.708 |
| Succinic acid | Organic Acids | -0.287 (-0.709, 0.135) | 0.181 |
| 2-Furoic acid | Organic Acids | -0.048 (-0.468, 0.371) | 0.821 |
| 2-Hydroxyglutaric acid | Organic Acids | -0.423 (-0.839, -0.007) | 0.046 |
| 3-Hydroxybutyric acid | Organic Acids | 0.267 (-0.152, 0.685) | 0.211 |
| 3-Methyl-2-oxopentanoic acid | Organic Acids | 0.412 (-0.006, 0.830) | 0.053 |
| Glycylproline | Peptides | 0.094 (-0.328, 0.517) | 0.660 |
| Homovanillic acid | Phenols | 0.144 (-0.278, 0.565) | 0.503 |
| 3,4-Dihydroxymandelic acid | Phenols | -0.072 (-0.491, 0.347) | 0.736 |
| Hydrocinnamic acid | Phenylpropanoic Acids | -0.123 (-0.543, 0.297) | 0.565 |
| Phenyllactic acid | Phenylpropanoic Acids | -0.029 (-0.448, 0.390) | 0.892 |
| 2-Hydroxycinnamic acid | Phenylpropanoic Acids | 0.017 (-0.403, 0.438) | 0.935 |
| Cinnamic acid | Phenylpropanoids | -0.166 (-0.583, 0.252) | 0.435 |
| Acetic acid | SCFAs | 0.119 (-0.300, 0.537) | 0.576 |
| Butyric acid | SCFAs | -0.216 (-0.633, 0.202) | 0.310 |
| Caproic acid | SCFAs | -0.126 (-0.545, 0.293) | 0.554 |
| Isobutyric acid | SCFAs | -0.133 (-0.552, 0.287) | 0.534 |
| Isocaproic acid | SCFAs | -0.284 (-0.701, 0.133) | 0.181 |
| Isovaleric acid | SCFAs | -0.032 (-0.451, 0.386) | 0.879 |
| Propionic acid | SCFAs | -0.239 (-0.656, 0.179) | 0.261 |
| Valeric acid | SCFAs | -0.040 (-0.461, 0.381) | 0.852 |

Adjusted for age and parity.

Table S2 The association between serum metabolites and GDM risk.

| Metabolite | Class | OR (95% CI) | P value |
| --- | --- | --- | --- |
| Acetylglycine | Amino Acids | 1.30 (0.97, 1.76) | 0.082 |
| Phenylacetylglutamine | Amino Acids | 0.79 (0.58, 1.06) | 0.125 |
| Aminocaproic acid | Amino Acids | 0.87 (0.66, 1.16) | 0.354 |
| Citrulline | Amino Acids | 1.40 (1.04, 1.91) | 0.028 |
| Creatine | Amino Acids | 0.88 (0.66, 1.16) | 0.362 |
| Dimethylglycine | Amino Acids | 1.69 (1.20, 2.51) | 0.005 |
| GABA | Amino Acids | 1.00 (0.73, 1.34) | 0.987 |
| Glycine | Amino Acids | 0.98 (0.74, 1.30) | 0.898 |
| Alanine | Amino Acids | 1.02 (0.77, 1.36) | 0.872 |
| alpha-Aminobutyric acid | Amino Acids | 1.23 (0.92, 1.66) | 0.165 |
| Arginine | Amino Acids | 1.19 (0.90, 1.60) | 0.227 |
| Asparagine | Amino Acids | 1.02 (0.76, 1.35) | 0.914 |
| Aspartic acid | Amino Acids | 1.13 (0.85, 1.52) | 0.407 |
| Glutamic acid | Amino Acids | 0.96 (0.72, 1.27) | 0.771 |
| Glutamine | Amino Acids | 1.16 (0.87, 1.56) | 0.319 |
| Histidine | Amino Acids | 1.05 (0.79, 1.40) | 0.729 |
| Homocitrulline | Amino Acids | 0.83 (0.61, 1.11) | 0.222 |
| Isoleucine | Amino Acids | 1.04 (0.78, 1.38) | 0.796 |
| Leucine | Amino Acids | 1.13 (0.85, 1.50) | 0.418 |
| Lysine | Amino Acids | 1.00 (0.75, 1.34) | 0.978 |
| Methionine | Amino Acids | 1.28 (0.96, 1.72) | 0.098 |
| Pipecolic acid | Amino Acids | 1.22 (0.91, 1.77) | 0.232 |
| Proline | Amino Acids | 1.10 (0.83, 1.47) | 0.504 |
| Serine | Amino Acids | 1.04 (0.78, 1.38) | 0.809 |
| Threonine | Amino Acids | 1.14 (0.86, 1.54) | 0.360 |
| Tryptophan | Amino Acids | 0.99 (0.74, 1.31) | 0.934 |
| Tyrosine | Amino Acids | 1.02 (0.77, 1.36) | 0.868 |
| Valine | Amino Acids | 1.22 (0.91, 1.63) | 0.183 |
| Methylcysteine | Amino Acids | 1.00 (0.75, 1.33) | 0.992 |
| N-Acetylaspartic acid | Amino Acids | 0.89 (0.67, 1.19) | 0.436 |
| N-Acetylglutamine | Amino Acids | 1.04 (0.78, 1.39) | 0.794 |
| N-Acetylserine | Amino Acids | 1.01 (0.76, 1.35) | 0.932 |
| Ornithine | Amino Acids | 1.17 (0.87, 1.57) | 0.299 |
| Benzenebutanoic acid | Benzenoids | 1.31 (0.97, 1.82) | 0.090 |
| Phenylpyruvic acid | Benzenoids | 0.87 (0.65, 1.16) | 0.348 |
| Hippuric acid | Benzoic Acids | 0.92 (0.68, 1.23) | 0.571 |
| Beta_Ursodeoxycholic Acid | Bile Acids | 1.07 (0.80, 1.46) | 0.639 |
| Chenodeoxycholic acid | Bile Acids | 1.35 (1.00, 1.90) | 0.065 |
| Cholic acid | Bile Acids | 1.14 (0.86, 1.58) | 0.386 |
| Deoxycholic acid | Bile Acids | 1.10 (0.82, 1.48) | 0.535 |
| Glycochenodeoxycholic acid | Bile Acids | 0.99 (0.74, 1.33) | 0.956 |
| Glycocholic acid | Bile Acids | 0.98 (0.73, 1.30) | 0.882 |
| Glycodeoxycholic acid | Bile Acids | 0.93 (0.69, 1.23) | 0.605 |
| Glycohyocholic acid | Bile Acids | 1.17 (0.88, 1.60) | 0.284 |
| Glycolithocholic acid | Bile Acids | 0.81 (0.60, 1.07) | 0.147 |
| Glycoursodeoxycholic acid | Bile Acids | 1.27 (0.95, 1.81) | 0.137 |
| Hyodeoxycholic acid | Bile Acids | 0.96 (0.72, 1.27) | 0.779 |
| isolithocholic acid | Bile Acids | 0.77 (0.55, 1.03) | 0.094 |
| Lithocholic acid | Bile Acids | 0.85 (0.63, 1.12) | 0.255 |
| Taurochenodeoxycholic acid | Bile Acids | 0.99 (0.74, 1.34) | 0.957 |
| Taurocholic acid | Bile Acids | 1.04 (0.78, 1.42) | 0.806 |
| Taurodeoxycholic acid | Bile Acids | 0.92 (0.68, 1.22) | 0.542 |
| Ursodeoxycholic acid | Bile Acids | 1.09 (0.82, 1.47) | 0.553 |
| Fructose | Carbohydrates | 0.95 (0.68, 1.28) | 0.697 |
| Gluconolactone | Carbohydrates | 0.92 (0.69, 1.22) | 0.557 |
| Glucose | Carbohydrates | 1.15 (0.86, 1.55) | 0.350 |
| Maltose | Carbohydrates | 0.79 (0.57, 1.06) | 0.125 |
| Xylose | Carbohydrates | 0.94 (0.69, 1.25) | 0.659 |
| Erythronic acid | Carbohydrates | 0.84 (0.62, 1.11) | 0.219 |
| Galactonic acid | Carbohydrates | 1.15 (0.86, 1.55) | 0.344 |
| Glyceric acid | Carbohydrates | 0.76 (0.56, 1.01) | 0.059 |
| Lactulose | Carbohydrates | 0.97 (0.73, 1.29) | 0.850 |
| N-Acetylglucosamine | Carbohydrates | 1.24 (0.93, 1.72) | 0.163 |
| N-Acetylneuraminic acid | Carbohydrates | 1.12 (0.84, 1.52) | 0.445 |
| Tartaric acid | Carbohydrates | 1.02 (0.76, 1.41) | 0.898 |
| Adipoylcarnitine | Carnitines | 1.15 (0.87, 1.54) | 0.334 |
| Carnitine | Carnitines | 0.73 (0.54, 0.98) | 0.038 |
| Decanoylcarnitine | Carnitines | 1.78 (1.27, 2.64) | 0.002 |
| Glutarylcarnitine | Carnitines | 1.05 (0.79, 1.41) | 0.734 |
| Hexanylcarnitine | Carnitines | 2.00 (1.33, 3.22) | 0.002 |
| Isovalerylcarnitine | Carnitines | 1.01 (0.76, 1.34) | 0.956 |
| Acetylcarnitine | Carnitines | 1.37 (1.02, 1.86) | 0.038 |
| Dodecanoylcarnitine | Carnitines | 1.60 (1.18, 2.22) | 0.003 |
| Linoleylcarnitine | Carnitines | 1.25 (0.94, 1.68) | 0.136 |
| Malonylcarnitine | Carnitines | 1.18 (0.88, 1.58) | 0.270 |
| Methylmalonylcarnitine | Carnitines | 1.24 (0.92, 1.67) | 0.160 |
| Tetradecanoylcarnitine | Carnitines | 1.42 (1.06, 1.94) | 0.022 |
| Octanoylcarnitine | Carnitines | 1.99 (1.35, 3.18) | 0.002 |
| Oleylcarnitine | Carnitines | 1.42 (1.06, 1.93) | 0.023 |
| Palmitoylcarnitine | Carnitines | 1.22 (0.91, 1.64) | 0.180 |
| Propionylcarnitine | Carnitines | 0.83 (0.62, 1.10) | 0.204 |
| Stearylcarnitine | Carnitines | 1.05 (0.79, 1.40) | 0.734 |
| 2-Methylbutyroylcarnitine | Carnitines | 1.27 (0.95, 1.73) | 0.116 |
| 3-Hydroxylisovalerylcarnitine | Carnitines | 0.99 (0.74, 1.33) | 0.953 |
| Adipic acid | Fatty Acids | 0.81 (0.60, 1.08) | 0.169 |
| Adrenic acid | Fatty Acids | 1.21 (0.91, 1.65) | 0.206 |
| alpha-Linolenic acid | Fatty Acids | 1.33 (1.00, 1.80) | 0.054 |
| Arachidonic acid | Fatty Acids | 1.02 (0.76, 1.35) | 0.911 |
| Azelaic acid | Fatty Acids | 0.90 (0.67, 1.20) | 0.485 |
| 2-Butenoic acid | Fatty Acids | 1.03 (0.77, 1.36) | 0.856 |
| Citraconic acid | Fatty Acids | 0.84 (0.60, 1.13) | 0.265 |
| Citramalic acid | Fatty Acids | 1.03 (0.77, 1.45) | 0.832 |
| Decanoic acid | Fatty Acids | 1.40 (1.02, 2.03) | 0.052 |
| DHA | Fatty Acids | 0.96 (0.72, 1.29) | 0.801 |
| DPAn-6 | Fatty Acids | 1.19 (0.89, 1.60) | 0.252 |
| DPA | Fatty Acids | 1.18 (0.89, 1.59) | 0.260 |
| Dodecanoic acid | Fatty Acids | 1.15 (0.86, 1.57) | 0.353 |
| EPA | Fatty Acids | 0.85 (0.60, 1.14) | 0.292 |
| gamma-Linolenic acid | Fatty Acids | 1.22 (0.92, 1.64) | 0.178 |
| Heptanoic acid | Fatty Acids | 1.11 (0.83, 1.58) | 0.495 |
| Itaconic acid | Fatty Acids | 0.99 (0.75, 1.32) | 0.956 |
| Linoleic acid | Fatty Acids | 1.48 (1.11, 2.00) | 0.010 |
| Methylglutaric acid | Fatty Acids | 1.07 (0.80, 1.43) | 0.663 |
| 9E-tetradecenoic acid | Fatty Acids | 1.16 (0.87, 1.57) | 0.330 |
| Myristic acid | Fatty Acids | 1.01 (0.76, 1.36) | 0.955 |
| Myristoleic acid | Fatty Acids | 1.17 (0.87, 1.58) | 0.296 |
| Nonanoic acid | Fatty Acids | 0.88 (0.65, 1.16) | 0.364 |
| Octanoic acid | Fatty Acids | 1.46 (1.04, 2.17) | 0.046 |
| Oleic acid | Fatty Acids | 1.57 (1.17, 2.14) | 0.003 |
| Palmitoleic acid | Fatty Acids | 1.26 (0.94, 1.71) | 0.122 |
| Pentadecanoic acid | Fatty Acids | 0.80 (0.31, 1.13) | 0.408 |
| Pimelic acid | Fatty Acids | 0.76 (0.55, 1.01) | 0.070 |
| Ricinoleic acid | Fatty Acids | 0.98 (0.74, 1.31) | 0.896 |
| Sebacic acid | Fatty Acids | 0.88 (0.66, 1.16) | 0.356 |
| Tridecanoic acid | Fatty Acids | 0.88 (0.50, 1.20) | 0.472 |
| Undecanoic acid | Fatty Acids | 1.21 (0.91, 1.62) | 0.198 |
| (10Z,13Z)-Nonadecadienoic acid | Fatty Acids | 1.36 (1.01, 1.86) | 0.048 |
| 10Z-Heptadecenoic acid | Fatty Acids | 1.32 (0.97, 1.86) | 0.094 |
| 2-Hydroxy-3-methylbutyric acid | Fatty Acids | 1.42 (1.06, 1.97) | 0.026 |
| 3-Hydroxyisovaleric acid | Fatty Acids | 0.94 (0.70, 1.24) | 0.650 |
| 3-Methyladipic acid | Fatty Acids | 0.84 (0.62, 1.12) | 0.246 |
| 5Z-Dodecenoic acid | Fatty Acids | 1.37 (1.02, 1.86) | 0.039 |
| Dihomo-gamma-linolenic acid | Fatty Acids | 1.28 (0.96, 1.74) | 0.095 |
| 9-Pentadecenoic acid | Fatty Acids | 0.86 (0.58, 1.15) | 0.345 |
| Indoleacetic acid | Indoles | 1.11 (0.83, 1.50) | 0.469 |
| Indole-3-propionic acid | Indoles | 0.87 (0.62, 1.15) | 0.345 |
| AMP | Nucleotides | 0.96 (0.72, 1.28) | 0.765 |
| alpha-Hydroxyisobutyric acid | Organic Acids | 1.24 (0.93, 1.65) | 0.143 |
| alpha-Ketoisovaleric acid | Organic Acids | 1.60 (1.19, 2.20) | 0.003 |
| Aconitic acid | Organic Acids | 1.29 (0.94, 1.93) | 0.163 |
| Citric acid | Organic Acids | 1.74 (1.29, 2.41) | 5.12e-04 |
| Fumaric acid | Organic Acids | 1.04 (0.79, 1.39) | 0.765 |
| Glutaconic acid | Organic Acids | 0.98 (0.73, 1.30) | 0.863 |
| Glutaric acid | Organic Acids | 0.92 (0.69, 1.22) | 0.577 |
| Glycolic acid | Organic Acids | 0.97 (0.73, 1.29) | 0.830 |
| Guanidoacetic acid | Organic Acids | 1.10 (0.83, 1.47) | 0.497 |
| Isocitric acid | Organic Acids | 1.31 (0.98, 1.78) | 0.071 |
| Ketoleucine | Organic Acids | 1.47 (1.09, 2.00) | 0.012 |
| Lactic acid | Organic Acids | 1.23 (0.92, 1.65) | 0.159 |
| Malic acid | Organic Acids | 1.18 (0.89, 1.58) | 0.257 |
| Maleic acid | Organic Acids | 0.88 (0.66, 1.17) | 0.385 |
| Methylmalonic acid | Organic Acids | 1.18 (0.88, 1.60) | 0.276 |
| Oxoadipic acid | Organic Acids | 0.96 (0.72, 1.27) | 0.753 |
| Oxoglutaric acid | Organic Acids | 0.97 (0.72, 1.30) | 0.854 |
| Pyruvic acid | Organic Acids | 0.84 (0.62, 1.11) | 0.224 |
| Succinic acid | Organic Acids | 1.19 (0.89, 1.60) | 0.247 |
| 2-Furoic acid | Organic Acids | 0.82 (0.41, 1.13) | 0.378 |
| 2-Hydroxyglutaric acid | Organic Acids | 0.93 (0.70, 1.23) | 0.613 |
| 3-Hydroxybutyric acid | Organic Acids | 1.43 (1.05, 2.03) | 0.034 |
| 3-Methyl-2-oxopentanoic acid | Organic Acids | 1.38 (1.03, 1.87) | 0.035 |
| Glycylproline | Peptides | 1.04 (0.78, 1.39) | 0.799 |
| Homovanillic acid | Phenols | 1.80 (1.16, 3.01) | 0.017 |
| 3,4-Dihydroxymandelic acid | Phenols | 1.01 (0.76, 1.34) | 0.959 |
| Hydrocinnamic acid | Phenylpropanoic Acids | 0.64 (0.33, 0.94) | 0.077 |
| Phenyllactic acid | Phenylpropanoic Acids | 1.03 (0.78, 1.38) | 0.817 |
| 2-Hydroxycinnamic acid | Phenylpropanoic Acids | 0.69 (0.15, 1.07) | 0.365 |
| Cinnamic acid | Phenylpropanoi Acids | 0.92 (0.69, 1.23) | 0.579 |
| Acetic acid | SCFAs | 1.11 (0.84, 1.49) | 0.462 |
| Butyric acid | SCFAs | 1.34 (1.01, 1.81) | 0.048 |
| Caproic acid | SCFAs | 1.01 (0.76, 1.35) | 0.940 |
| Isobutyric acid | SCFAs | 1.05 (0.79, 1.41) | 0.726 |
| Isocaproic acid | SCFAs | 0.84 (0.63, 1.12) | 0.239 |
| Isovaleric acid | SCFAs | 0.90 (0.67, 1.19) | 0.448 |
| Propionic acid | SCFAs | 0.99 (0.75, 1.32) | 0.972 |
| Valeric acid | SCFAs | 0.97 (0.73, 1.29) | 0.820 |

Adjusted for age and parity.

Table S3 The mediating role of individual and LVs metabolites in early-mid pregnancy in the association between pBMI and GDM risk

| Mediator | Direct effect (95% CI) | Indirect effect (95% CI) | P for indirect effect | Mediated proportion (%) |
| --- | --- | --- | --- | --- |
| Metabolites |  |  |  |  |
| Hexanylcarnitine | 0.0039(0.0007-0.0089) | 0.0013(0.0001-0.0054) | **0.014** | **19.5** |
| Octanoylcarnitine | 0.0040(0.0007-0.0089) | 0.0013(0.0001-0.0053) | **0.014** | **19.6** |
| Decanoylcarnitine | 0.0044(0.0008-0.0096) | 0.0010(0.0000-0.0043) | **0.042** | **14.1** |
| 2-Hydroxy-3-methylbutyric acid | 0.0045(0.0009-0.0097) | 0.0008(-0.0001-0.0035) | 0.104 | 10.1 |
| Linoleic acid | 0.0046(0.0008-0.0100) | 0.0006(-0.0000-0.0030) | 0.072 | 8.8 |
| Citric acid | 0.0038(0.0007-0.0084) | 0.0014(0.0001-0.0057) | **0.006** | **19.6** |
| a-Ketoisovaleric acid | 0.0042(0.0008-0.0090) | 0.0011(0.0000-0.0046) | **0.012** | **15.1** |
| Ketoleucine | 0.0045(0.0009-0.0095) | 0.0008(-0.0000-0.0038) | 0.066 | 11.1 |
| LVs |  |  |  |  |
| LV1 | 0.0035(0.0006-0.0078) | 0.0017(0.0001-0.0068) | **0.004** | **24.0** |
| LV2 | 0.0039(0.0007-0.0088) | 0.0013(0.0001-0.0053) | **0.008** | **19.1** |

LV, latent variable. Mediation models were adjusted for age and parity

Table S4 The serial mediated effect of LVs in the association between pBMI and GDM risk

|  | b (95% CI) | P value | Mediated proportion (%) |
| --- | --- | --- | --- |
| LV1→LV2 |  |  |  |
| Direct effect | 0.024 (0.002, 0.045) | 0.026 |  |
| Indirect effect |  |  |  |
| LV1 | 0.008 (0.001, 0.016) | **0.031** | **21.1%** |
| LV2 | 0.004 (-0.001, 0.010) | 0.171 | 10.5% |
| LV1-LV2 | 0.002 (0.000, 0.004) | **0.049** | **5.3%** |
| LV2→LV1 |  |  |  |
| Direct effect | 0.024 (0.002, 0.045) | 0.026 |  |
| Indirect effect |  |  |  |
| LV2 | 0.006 (0.001, 0.012) | 0.065 | 15.8% |
| LV1 | 0.007 (0.001, 0.013) | **0.041** | **18.4%** |
| LV2-LV1 | 0.001 (0.000, 0.003) | 0.076 | 2.6% |

LV, latent variable. Mediation models were adjusted for age and parity
